# Complete Genome Sequences of Bacillus Phages Janet and OTooleKemple52

**DOI:** 10.1128/genomeA.00083-18

**Published:** 2018-05-10

**Authors:** Brenna Kent, Thomas Raymond, Philip D. Mosier, Allison A. Johnson

**Affiliations:** a Center for the Study of Biological Complexity, Virginia Commonwealth University, Richmond, Virginia, USA; b Department of Medicinal Chemistry and Institute for Structural Biology, Drug Discovery and Development, School of Pharmacy, Virginia Commonwealth University, Richmond, Virginia, USA

## Abstract

We report here the genome sequences of two novel Bacillus cereus group-infecting bacteriophages, Janet and OTooleKemple52. These bacteriophages are double-stranded DNA-containing Myoviridae isolated from soil samples. While their genomes share a high degree of sequence identity with one another, their host preferences are unique.

## GENOME ANNOUNCEMENT

The bacteriophages Janet and OTooleKemple52 (OTK52) were isolated from soil samples collected in 2016 from Poznań, Poland (global positioning system [GPS] coordinates 52.458737°N, 16.910597°E) and Chesapeake, VA (GPS coordinates 36.696529°N, 76.243623°W), respectively, as part of the SEA-PHAGES program (1). These bacteriophages were isolated after soil enrichment with Bacillus thuringiensis serovar kurstaki (Btk). When isolated, Janet formed clear round plaques ranging from 2 to 4 mm in diameter, while OTK52 formed 3-mm plaques with halos on lawns of Btk. The phages were tested for their ability to form plaques on a panel of nine Bacillus species and strains. Janet was also able to form plaques on B. thuringiensis serovar konkukian, B. thuringiensis serovar israelensis, and B. thuringiensis 350, suggesting a preference for infecting B. thuringiensis strains, while OTK52 could infect only two bacteria, B. thuringiensis 350 and Bacillus cereus 23857, indicating a relatively narrow host range. Transmission electron microscopy and genome analysis support a Myoviridae morphology for both phages.

Phage genomic DNA was sequenced by the Pittsburgh Bacteriophage Institute using Illumina sequencing. The assembly program Newbler was used to produce a single contig from 50,000 reads, resulting in ∼100-fold depth of coverage. Gene annotation was completed using DNA Master (see http://cobamide2.bio.pitt.edu/computer.htm), which includes the open reading frame (ORF) prediction tools GeneMark (2) and Glimmer (3) and the tRNA prediction tool ARAGORN (4). Functional analysis was performed using BLASTp (5) and HHpred (6).

Janet has a length of 160,705 bp, with terminal repeats of 2,434 bp, while OTooleKemple52 (OTK52) has a genome length of 161,807 bp, with terminal repeats of 2,426 bp. The genomes have 38% GC content. Janet and OTK52 are classified as Myoviridae and are part of the C2 cluster for Bacillus phages (7). These phages share 95% query coverage and 96 to 97% identity with each other, as well as similarly high values to five other cluster C2 Bacillus phages in GenBank. The best BLAST matches for Janet and OTK52 are Bacillus phages CAM003, HoodyT, and Bastille (accession numbers KJ489397, KJ489400, and JF966203, respectively). It is interesting to note that varied plaque morphology is observed in other C2 phages, where CAM003 and Evoli formed clear plaques and HoodyT formed cloudy plaques on the same host bacteria (http://bacillus.phagesdb.org).

Janet has 286 open reading frames and seven genes encoding tRNAs, while OTK52 has 291 open reading frames plus seven genes encoding tRNAs. Typically, C2 cluster phages contain an average of 296 genes, of which eight genes code for tRNAs. Also, 269 proteins (92%) are from identical families, supporting the high degree of relatedness of this genome cluster. Each genome contains two novel genes without homologs in the NCBI nonredundant database. We predicted functions for almost 20% of the ORFs (43 and 41 protein-coding ORFs from Janet and OTK52, respectively), with most of those functional annotations being structural, lysis, and DNA-modifying genes.

### Accession number(s).

The complete genome sequences of the Bacillus phages Janet and OTooleKemple52 are available in GenBank under accession numbers MF288922 and MF288921, respectively.
